# Data on thermostable β-glucosidase immobilized by Zn^2+^

**DOI:** 10.1016/j.dib.2018.03.105

**Published:** 2018-03-27

**Authors:** Xuejia Shi, Linguo Zhao, Jianjun Pei, Lin Ge, Pengwei Wan, Zhenzhong Wang, Wei Xiao

**Affiliations:** a Co-Innovation Center for Sustainable Forestry in Southern China, Nanjing Forestry University, 159 Long Pan Road, Nanjing 210037, China; b College of Chemical Engineering, Nanjing Forestry University, 159 Long Pan Road, Nanjing 210037, China; c Jiangsu Key Lab for the Chemistry & Utilization of Agricultural and Forest Biomass, 159 Long Pan Road, Nanjing 210037, China; d Jiangsu Kanion Pharmaceutical Co., Ltd., 58 Haichang South Road, Lianyungang 222001, Jiangsu Province, China

## Abstract

In this article, the methods for detection of enzyme activity and protein concentration are described. The data of the calibration curves can be used for a further understanding on the assays of enzyme activity measured with *p*-nitrophenyl-β-D-glucopyranoside (*p*NPG) or cellobiose as the substrate. In addition, the data presented provides an analytic method for measuring protein concentration in mixed samples.

**Specifications table**

**Table:** 

Subject area	Biology
More specific subject area	Enzyme Eng&Proteins
Type of data	Figure
How data was acquired	Using an absorbance microplate reader (SpectraMax190, Molecular Devices, LLC, Sunnyvale, CA)
Data format	Raw and analyzed data
Experimental factors	Assays of enzyme activity and protein concentration
Experimental features	The calibration curves of enzyme activity and protein concentration were offered
Data source location	Nanjing, China
Data accessibility	The data are available with this article

**Value of the data**

1. The data makes available to the detection of enzyme activity with *p*NPG as the substrate.
2. The data will guide the assay of enzyme activity for immobilized enzyme with cellobiose as the substrate.
3. The data presented can provide a calibration curve for measuring protein concentration in mixed samples.

## 1 Data

**Fig. 1:**
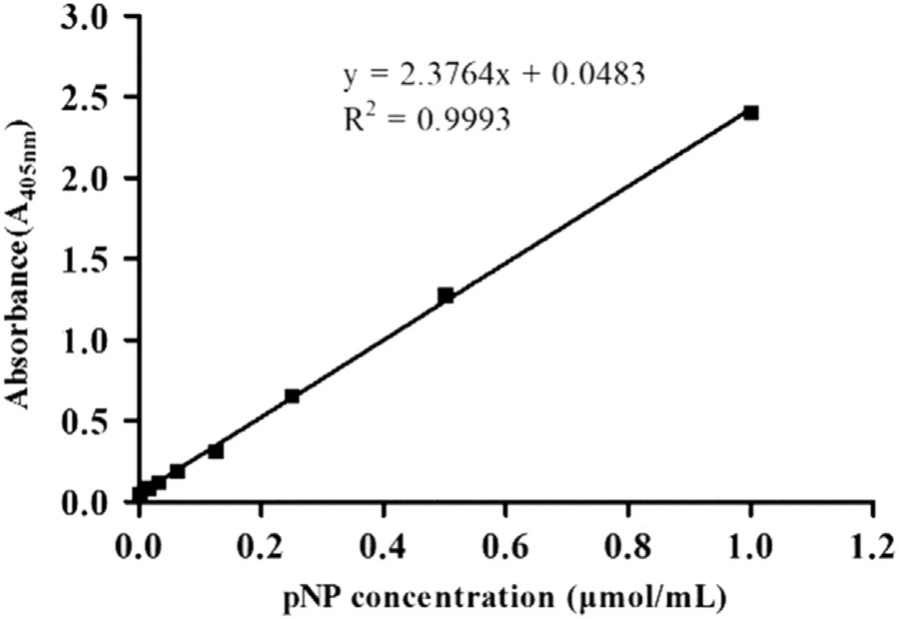
The calibration curve about assay of enzyme activity with *p*NPG as the substrate.

**Fig. 2:**
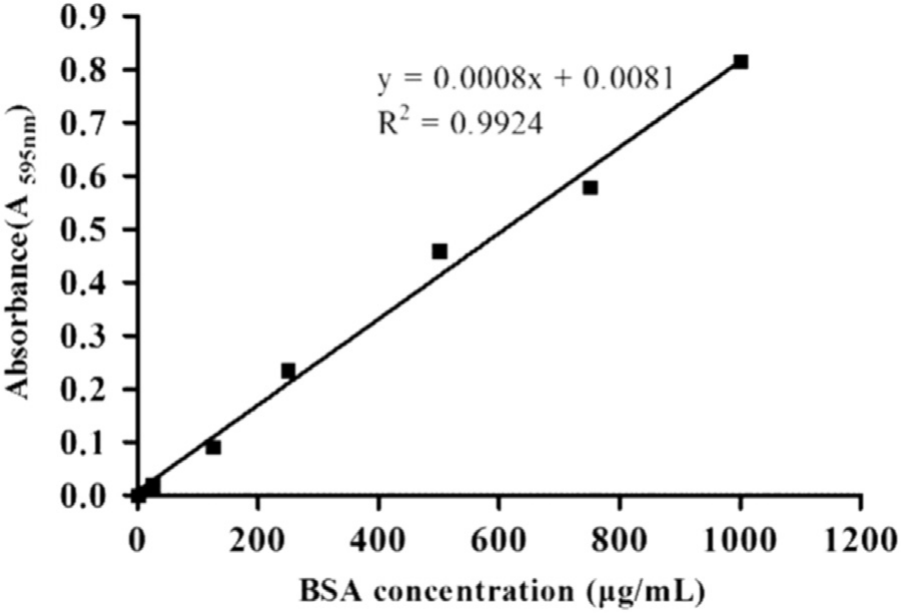
The calibration curve about detection of protein concentration with bovine serum albumin (BSA) as the reference.

The calibration curve about the assay of enzyme activity measured with *p*NPG as substrate is given in Fig. 1. Glucose Assay Kit purchased from Shanghai Rongsheng Biological Technology Co., Ltd provides the calibration curve about the assay of enzyme activity with cellobiose as substrate. Meanwhile, Fig. 2 showed a calibration curve for the detection of protein concentration in mixed samples.

## 2 Experimental design, materials and methods

Enzyme activity of immobilized enzyme required was measured with *p*NPG as the substrate [1]. The pNP (0, 0.015, 0.030,0.0625, 0.125, 0.25, 0.50, 1.00 μmol/mL) was added to 1.5 mL tube containing 200 μL citric acid Na_2_HPO_4_ buffer (100 mM) and 600 μL Na_2_CO_3_ (1 M), The calibration curve was given by: y = 2.3764x + 0.0483 in Fig. 1, where x is the *p*NP concentration (μmol/mL) and , y is the absorbance of A405nm. Then the absorbance is calculated as enzyme activity from working curve. Enzyme activity of enzyme required was measured by cellobiose as the substrate. The calibration curve was provided by Glucose Assay Kit purchased from Shanghai Rongsheng Biological Technology Co., Ltd (Shanghai, China). And the calibration curve was given by:

$$y=\frac{A_{1}}{A_{0}}\times B$$

where y is the glucose concentration (mmol/L); A_0_ is the absorbance of A_505nm_ from standard sample; A_1_ is the absorbance of A_505nm_ from sample detected; B represented the concentration of standard sample (mmol/L). Then the absorbance is calculated as enzyme activity from working curve.

Protein concentration was detected by Bradford protein Assay Kit and the bovine serum albumin (BSA) was as the reference [2]. The mixture contained 200 μL Bradford protein Assay Kit and BSA (0, 25, 125, 250, 500, 750, and 1000 μg/mL) which was dissolved in deionized water. The calibration curve was given by: y = 0.0008x + 0.0081 in Fig. 2, where x is the BSA concentration (μg/mL), y is the absorbance of A_595nm_. Then the absorbance is calculated as protein concentration from working curve.
